# Unraveling the role of STAT3 in Cancer Cachexia: pathogenic mechanisms and therapeutic opportunities

**DOI:** 10.3389/fendo.2025.1608612

**Published:** 2025-07-09

**Authors:** Xinyi Lv, Shengguang Ding

**Affiliations:** ^1^ Department of Thoracic Surgery, Affiliated Hospital 2 of Nantong University, Nantong First People’s Hospital, Nantong, China; ^2^ School of Medicine, Nantong University, Nantong, China

**Keywords:** cancer cachexia, STAT3, systemic inflammation, muscle wasting, pathogenic mechanisms, therapeutic strategies

## Abstract

Cancer cachexia is a complex, multifactorial syndrome characterized by severe weight loss, muscle wasting, and systemic inflammation, significantly contributing to cancer-related morbidity and mortality. Signal transducer and activator of transcription 3 (STAT3) has emerged as a central mediator in the pathogenesis of this multifactorial condition. STAT3 regulates a broad range of cellular processes including inflammation, proteolysis, and mitochondrial dysfunction across multiple tissues, particularly skeletal muscle and adipose tissue. Persistent activation of STAT3 in response to tumor-derived and host-derived cytokines drives catabolic signaling cascades, disrupts anabolic pathways, and impairs energy homeostasis. Recent studies have illuminated the cross-talk between STAT3 and other signaling pathways that exacerbate cachexia-related metabolic imbalances. These findings position STAT3 not only as a critical mediator of cachexia progression but also as a promising therapeutic target. Pharmacological inhibition of STAT3 signaling has demonstrated efficacy in preclinical models, offering potential avenues for clinical intervention. This review provides a comprehensive overview of the molecular mechanisms by which STAT3 contributes to cancer cachexia and discusses emerging therapeutic strategies aimed at modulating STAT3 activity to mitigate the progression of this debilitating syndrome.

## Introduction

Cancer cachexia is a multifactorial syndrome characterized by severe body weight loss, muscle atrophy, anorexia, and fatigue, which cannot be fully reversed through conventional nutritional support or pharmacological interventions (1, 2). This condition arises from a complex interplay of metabolic dysfunction and inflammatory mediators, including elevated levels of pro-inflammatory cytokines such as interleukin-6 (IL-6) and tumor necrosis factor-alpha (TNF-α), increased energy expenditure, and tumor-derived factors that accelerate muscle and fat wasting (3, 4). Affecting up to 80% of patients in advanced cancer stages, cachexia significantly contributes to morbidity and mortality by impairing physical function, reducing treatment tolerance, and altering drug metabolism due to progressive muscle depletion and overall physiological decline (5–7). The associated symptoms-weight loss, muscle wasting, appetite loss, fatigue, and diminished quality of life-underscore the urgent need for effective management strategies. A multidisciplinary approach integrating nutritional support, pharmacological therapies such as anti-inflammatory agents and appetite stimulants, and structured physical exercise is essential to mitigating muscle loss and improving functional outcomes (8). Given the profound impact of cancer cachexia on patient survival and treatment efficacy, advancing the understanding of its complex etiology remains critical for developing more targeted and effective therapeutic interventions.

The mammalian Signal Transducer and Activator of Transcription (STAT) family comprises STAT1, STAT2, STAT3, STAT4, STAT5a, STAT5b, and STAT6, all of which mediate critical intracellular signaling pathways (9). Among these, STAT3 serves as a key transcription factor regulating diverse cellular processes, including cell growth (10, 11), apoptosis (12), and immune response modulation (13, 14). Notably, STAT3 is integral to both inflammation and cancer progression, as its persistent activation is frequently observed in various malignancies, where it drives tumorigenesis by promoting cell proliferation, inhibiting apoptosis, and facilitating angiogenesis (15, 16). Beyond oncogenesis, STAT3 plays a central role in immune regulation, particularly through the modulation of pro-inflammatory cytokines and the differentiation of Th17 cells (17–19), further linking it to chronic inflammation and autoimmune disorders (20, 21). Clinically, aberrant STAT3 signaling is implicated in multiple diseases, with constitutive activation often correlating with poor prognosis in cancer due to its involvement in sustaining tumor growth and survival (17, 21). Given its broad impact, STAT3 has emerged as a prominent therapeutic target, prompting extensive research into the development of STAT3 inhibitors and RNA interference strategies aimed at mitigating its pathological activity (22–24). These ongoing efforts underscore the significance of STAT3 in both physiological and disease contexts, highlighting its potential as a target for novel therapeutic interventions.

STAT3’s involvement in cancer cachexia is primarily driven by its role in mediating inflammatory responses (25) and its contribution to muscle wasting and dysregulated fat metabolism (26, 27). Elevated levels of pro-inflammatory cytokines, such as IL-6 and TNF-α, which are commonly observed in cancer cachexia, trigger STAT3 activation (27, 28), leading to the transcription of genes that exacerbate systemic inflammation, accelerate muscle protein degradation, and disrupt lipid metabolism (1, 29, 30). Furthermore, STAT3 facilitates the interaction between cancer cells and the host immune system, amplifying cachexia’s systemic effects by fostering immunosuppression and promoting tumor progression (17, 31). This interplay between chronic inflammation, metabolic dysfunction, and immune dysregulation underscores STAT3’s pivotal role in cancer cachexia pathogenesis. Consequently, a deeper understanding of STAT3’s function in this condition is essential for developing targeted therapeutic strategies aimed at mitigating its debilitating effects, improving patient outcomes, and enhancing quality of life.

## Overview of STAT3 activation pathways

STAT3 is a pivotal transcription factor that regulates diverse cellular functions, including proliferation, differentiation, survival, inflammation, and immune response (32, 33). STAT3 activation occurs primarily through cytokine and growth factor signaling, with the canonical pathway involving tyrosine phosphorylation (33). In this mechanism, cytokines from the interleukin-6 (IL-6) family (e.g., IL-6, IL-11, IL-31, leukemia inhibitory factor [LIF], oncostatin M [OSM], ciliary neurotrophic factor [CNTF], and cardiotrophin-1 [CT-1]), IL-10 family cytokines (IL-10, IL-19, IL-20, IL-22, IL-24, and IL-26), growth factors such as epidermal growth factor (EGF) and platelet-derived growth factor (PDGF), and interferons (IFNs) (9, 32, 34–36) bind to their respective receptors, triggering intracellular kinase activation. Janus kinases (JAK1, JAK2, TYK2) and receptor tyrosine kinases (EGFR, PDGFR, FGFR) phosphorylate STAT3 at Tyr705, inducing dimerization via SH2-domain interactions (32, 37, 38). The activated STAT3 dimer then translocates to the nucleus, where it binds to specific STAT-binding elements (SBEs) in gene promoters, regulating genes associated with survival (Bcl-xL), proliferation (Cyclin D1, c-Myc), inflammation (IL-6, COX2), metastasis (MMPs), angiogenesis (VEGF), and immune evasion (14, 39–43). This tightly regulated signaling cascade underscores STAT3’s pivotal role in cellular homeostasis and disease pathology.

**Figure 1:**
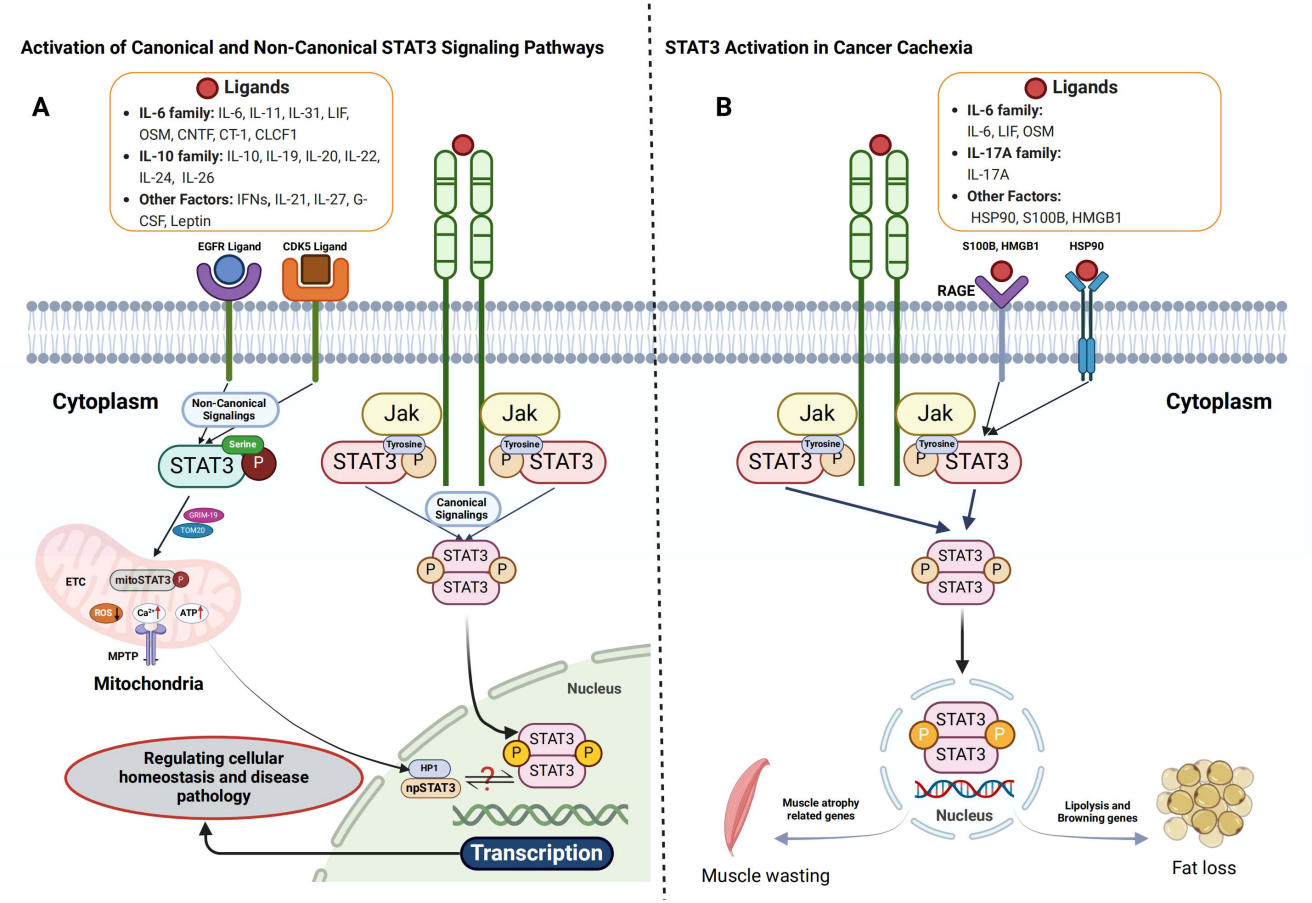
Activation of STAT3 signaling pathways and their role in cancer cachexia. **(A)** Both canonical and non-canonical STAT3 signaling pathways are crucial for cellular signaling. In the canonical pathway, cytokines such as IL-6, LIF, and OSM bind to their receptors, inducing receptor dimerization and the recruitment of JAKs. This interaction results in receptor phosphorylation at specific tyrosine residues, creating docking sites for STAT3. STAT3 is then phosphorylated by JAKs, dissociates from the receptor, forms homodimers or heterodimers, and translocates to the nucleus to regulate gene transcription. In contrast, the non-canonical STAT3 pathway involves mitochondrial STAT3 (mtSTAT3), unphosphorylated STAT3, and serine 727-phosphorylated STAT3 (p-STAT3 Ser727), alone or in combination with tyrosine 705-phosphorylated STAT3 (p-STAT3 Tyr705). These variants play a role in mitochondrial function, emphasizing STAT3’s involvement beyond transcriptional regulation. **(B)** In cancer cachexia, STAT3 activation occurs through both receptor- and non-receptor-mediated mechanisms. Pro-inflammatory cytokines or growth factors bind to cell surface receptors, leading to tyrosine phosphorylation, which facilitates the recruitment of JAKs or direct binding of STAT3 via its Src homology 2 (SH2) domain. Phosphorylated STAT3 dimerizes and enters the nucleus, promoting the transcription of genes involved in catabolic processes, driving tissue degradation and metabolic imbalance.

Beyond the canonical pathway, STAT3 activation also occurs through non-canonical mechanisms involving alternative post-translational modifications and extranuclear functions (44, 45). One such mechanism is serine phosphorylation, in which kinases like cyclin-dependent kinase 5 (CDK5) and EGFR phosphorylate STAT3 at Ser727, thereby enhancing its transcriptional activity and modulating mitochondrial function (45, 46). Additionally, a mitochondrial variant of STAT3 (mitoSTAT3) localizes to mitochondria, interacting with electron transport chain (ETC) complexes I and II to regulate ATP production and reactive oxygen species (ROS) generation (45). This function facilitates metabolic adaptation and promotes cancer cell survival (44, 47, 48). Moreover, non-phosphorylated STAT3 (npSTAT3) is implicated in cytoplasmic processes such as microtubule stabilization and protein degradation, demonstrating its diverse functional repertoire beyond transcriptional regulation (45, 49), as detailed in **Figure 1A**.

## STAT3 activation in cancer cachexia

Cancer cachexia, a debilitating syndrome characterized by muscle and adipose tissue wasting, is driven by elevated levels of pro-inflammatory cytokines, including IL-6-type cytokines (IL-6, LIF, OSM) (15, 25, 50), IL-17A (51), tumor necrosis factor-alpha (TNF-α), and interleukin-1 beta (IL-1β) (52, 53). Among these, IL-6 plays a central role in activating the JAK/STAT3 signaling pathway, a key mediator of systemic inflammation (54–56). Upon IL-6 binding to its receptor, JAK kinases phosphorylate STAT3, leading to dimerization and nuclear translocation (43). Within the nucleus, phosphorylated STAT3 regulates genes associated with inflammation, muscle protein degradation, and adipose tissue loss, exacerbating the imbalance between muscle synthesis and degradation that characterizes cachexia (54, 56–58). Correspondingly, cytokines such as OSM (50), IL-17A (51), and LIF (59) exploit STAT3 signaling to mediate muscle atrophy in cachectic models.

In addition to cytokine-driven activation, tumor-derived cachexia-inducing factors [such as IL-6, LIF, and G-CSF (59, 60)] and immune cells within the tumor microenvironment secrete active molecules that further enhance STAT3 activation, thereby promoting muscle atrophy. Among these factors, heat shock protein 90 (HSP90), receptor for advanced glycation end-products (RAGE) ligands, and S100B have emerged as novel contributors to muscle degradation. These factors induce muscle wasting through the p38 mitogen-activated protein kinase (MAPK)/myogenin axis (61) and STAT3 signaling (61, 62). Furthermore, receptor tyrosine kinases (RTKs), such as EGFR (63, 64), and metabolic regulators like leptin (65, 66) activate STAT3 via their respective homologous receptors, further implicating STAT3 in cachexia pathology. This intricate signaling network highlights the potential of STAT3 as a therapeutic target in cachexia management (**Figure 1B**). Future research focusing on STAT3 modulation could provide novel insights into intervention strategies, ultimately improving patient outcomes and quality of life.

## Mechanistic insights into STAT3 in cancer cachexia

**Figure 2:**
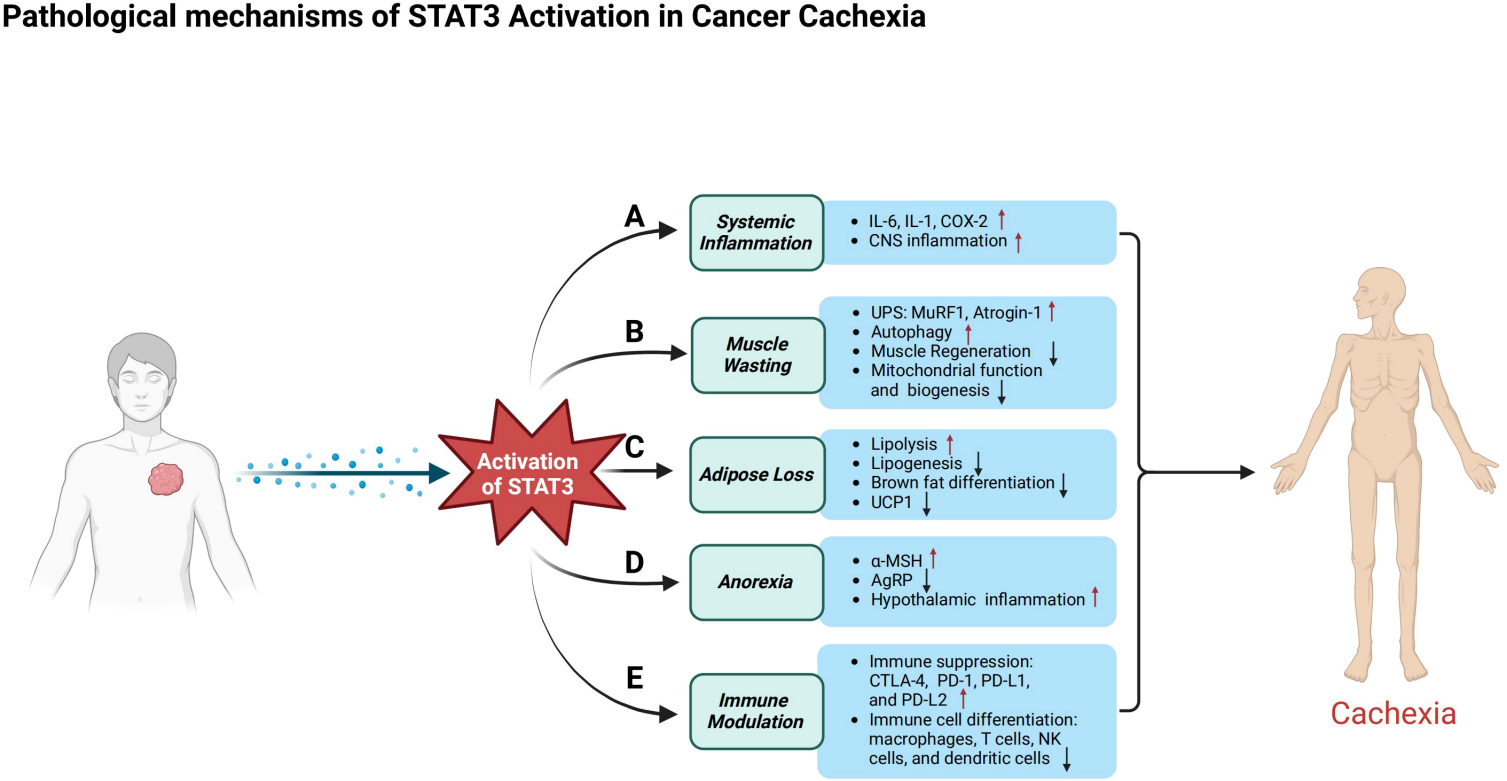
Pathological mechanisms of STAT3 in cancer cachexia. This figure illustrates the diverse roles of STAT3 activation in cancer patients, emphasizing its contribution to systemic inflammation, muscle atrophy, metabolic dysfunction, appetite regulation, and immune suppression. **(A)** In systemic inflammation, activated STAT3 triggers the release of pro-inflammatory cytokines such as IL-6, IL-1, and COX-2, exacerbates inflammation in the central nervous system, and contributes to anorexia. **(B)** In skeletal muscle, STAT3 activation induces atrophy by stimulating the ubiquitin–proteasome system and autophagy-related pathways, while impairing muscle regeneration by disrupting the differentiation, proliferation, and self-renewal of muscle satellite cells. Additionally, it compromises mitochondrial function. **(C)** In adipose tissue, activated STAT3 promotes lipolysis and metabolic dysregulation, inhibits lipogenesis, and suppresses brown adipose tissue differentiation and the expression of uncoupling protein 1 (UCP1). **(D)** In appetite regulation, STAT3 enhances the activity of pro-opiomelanocortin (POMC) neurons, increasing α-melanocyte-stimulating hormone (α-MSH) production to promote satiety, while simultaneously suppressing agouti-related peptide (AgRP) neurons that normally stimulate hunger, leading to reduced food intake and body weight loss. **(E)** STAT3 contributes to immune suppression by upregulating immune checkpoint molecules such as CTLA-4, PD-1, PD-L1, and PD-L2. Its activation alters the differentiation and function of immune cells, including macrophages, T cells, natural killer (NK) cells, and dendritic cells, reshaping the immune microenvironment and accelerating tumor progression.

**Figure 3:**
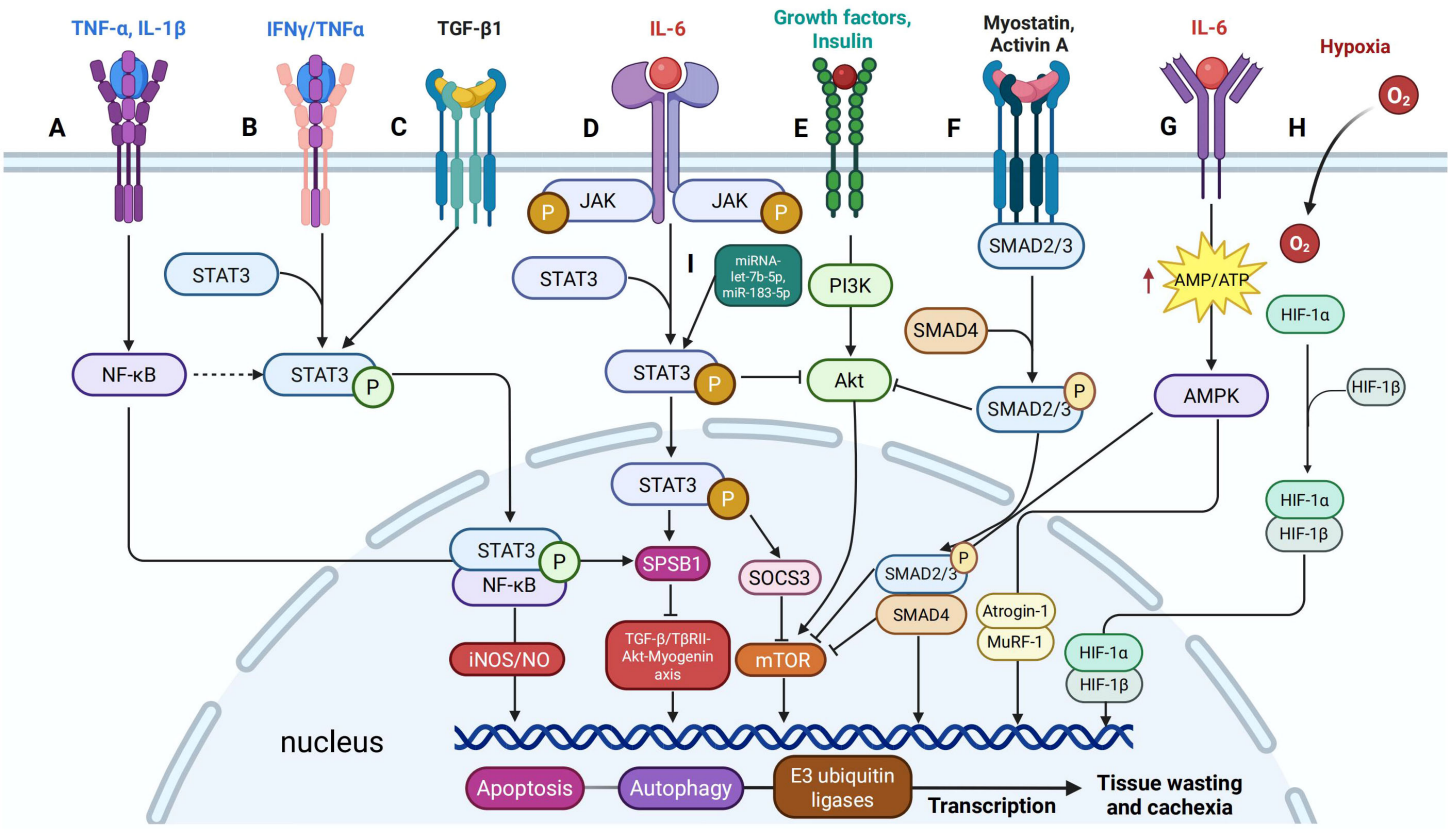
Signaling cross-talk between STAT3 and other pathways in cancer cachexia. **(A)** IFNγ/TNFα signaling induces phosphorylation of STAT3 at Y705, promoting its interaction with NF-κB to form a nuclear complex that activates the iNOS/NO pathway, a critical mediator of muscle loss. **(B)** TNF-α and IL-1β upregulate SOCS-box protein 1 (SPSB1) expression through NF-κB signaling. **(C)** IL-6 enhances SPSB1 expression via the glycoprotein 130/JAK2/STAT3 pathway, while TGF-β activates STAT3 in a SMAD-dependent manner. **(D)** TGFβ1 induces Tyr705 phosphorylation of STAT3 in C2C12 cells. **(E)** STAT3 signaling interacts with the PI3K/Akt/mTOR pathway by suppressing p-Akt activity. **(F)** Myostatin and Activin A activate SMAD2/3 signaling similarly and inhibit the insulin/IGF-1/Akt/mTOR pathway, reducing muscle mass and function. **(G)** IL-6 increases AMPK activity in C2C12 cells and mouse cancer cachexia models, and AMPK activation enhances myofibrillar protein degradation. **(H)** HIF-1α shifts muscle metabolism by upregulating glycolysis and downregulating oxidative phosphorylation. **(I)** The role of miRNAs in regulating STAT3 activation and enhancing its cachectic effects.

As previously mentioned, pro-inflammatory cytokines such as IL-6 activate STAT3, which subsequently induces the expression of atrogenes, including atrogin-1 and MuRF1. These atrogenes play a key role in promoting muscle proteolysis and impeding muscle regeneration by inhibiting satellite cell differentiation. In addition to its effects on muscle tissue, STAT3 also contributes to adipose tissue loss by upregulating lipolysis and suppressing lipogenesis. Furthermore, STAT3 facilitates the browning of white adipose tissue, leading to increased energy expenditure. This multifaceted role of STAT3 encompasses the regulation of protein and lipid metabolism, appetite control, and tumor immune responses. In the following sections, we will explore the key functions of STAT3 (**Figure 2**) and its cross-talk with other signaling pathways involved in these processes (**Figure 3**).

### STAT3 and systemic inflammation

Chronic inflammation, sustained by elevated levels of IL-6 and other pro-inflammatory cytokines, perpetuates persistent activation of STAT3, establishing a self-reinforcing cycle that drives the progression of cancer cachexia (14, 15, 29, 30). STAT3 activation enhances the transcription of inflammatory mediators such as IL-6, IL-1β, and COX-2, further amplifying cytokine production and systemic inflammation (14, 17, 30, 67). This pro-inflammatory feedback loop is compounded by multiple cytokines, including IL-6, leukemia inhibitory factor (LIF), and oncostatin M (OSM) (50, 56, 58, 68), as well as IFN-γ, TNF-α (69), and IL-17A (51), which converge on the STAT3 pathway, ensuring its sustained activation and contributing to metabolic dysregulation. While acute exposure to IL-6 and LIF can transiently promote protein synthesis via STAT3 and the Akt-mTORC1 pathway, it also induces SOCS3 expression to limit cytokine signaling (70). However, in chronic inflammatory states, prolonged STAT3 activity overrides this regulatory feedback, promoting muscle catabolism and systemic metabolic imbalance (68). Importantly, the influence of STAT3 extends beyond peripheral tissues. Neuroinflammatory signaling impacts the hypothalamus, disrupting appetite regulation by impairing orexigenic pathways and exacerbating anorexia, one of the cardinal features of cancer cachexia (25, 71, 72). This highlights the multifactorial nature of STAT3’s role, linking peripheral inflammation to central behavioral and metabolic dysfunction.

STAT3 also operates in concert with other inflammatory signaling cascades that further disrupt metabolic homeostasis. For instance, IFN-γ and TNF-α promote STAT3 phosphorylation at Y705, facilitating its interaction with NF-κB and forming a nuclear complex that induces the iNOS/NO pathway, a key contributor to inflammation-induced muscle wasting (69) (**Figure 3A**). Simultaneously, TNF-α and IL-1β activate NF-κB-dependent transcription of SOCS-box protein 1 (SPSB1) (**Figure 3B**), while IL-6 induces SPSB1 via the gp130/JAK2/STAT3 axis, collectively impairing myogenic differentiation and enhancing proteolytic activity through the ubiquitin–proteasome system (73, 74) (**Figure 3D**). Although NF-κB can inhibit apoptosis in some contexts, its chronic activation sustains oxidative stress and promotes muscle degradation (75). Furthermore, STAT3 impairs anabolic signaling by upregulating SOCS3, which suppresses the PI3K/Akt/mTOR pathway (**Figure 3E**). This inhibition reduces protein synthesis and exacerbates proteolysis, intensifying muscle atrophy (30, 76). These interactions position STAT3 as a central mediator linking inflammatory signaling to disrupted muscle metabolism.

In parallel, circulating cytokines influence AMP-activated protein kinase (AMPK), a key regulator of energy homeostasis. IL-6 increases AMPK activity in C2C12 cells and murine cachexia models, contributing to catabolic signaling (77, 78) (**Figure 3G**). In contrast, exercise training during early cachexia attenuates AMPK activation, restoring mTOR signaling and anabolic balance (78). Conversely, TNF-α suppresses AMPK via TNFR1 engagement, exacerbating metabolic dysfunction (79). Notably, chronic AMPK activation enhances protein degradation through both the autophagy–lysosome pathway and the ubiquitin–proteasome system (80–82). Collectively, these mechanisms form an intricate inflammatory-metabolic axis that drives the relentless tissue wasting characteristic of cancer cachexia.

### STAT3 in muscle wasting

Muscle wasting, a hallmark of cancer cachexia, is closely associated with aberrant activation of the STAT3 signaling pathway, which exerts multifaceted effects on muscle cell physiology and pathology (83, 84). STAT3 promotes muscle atrophy by mediating crosstalk between C/EBPδ and myostatin pathways, thereby repressing muscle growth and enhancing catabolic signaling (83). This leads to the upregulation of muscle-specific E3 ubiquitin ligases, including MuRF1 and Atrogin-1, which are central components of the ubiquitin–proteasome system responsible for proteolytic degradation of myofibrillar proteins (76, 85). In addition to proteasomal degradation, STAT3 contributes to autophagy-mediated muscle catabolism. Both nuclear and cytoplasmic STAT3 modulate autophagy-related processes, and its inhibition has been shown to restore autophagic flux by upregulating autophagy-related genes and altering eukaryotic initiation factor 2α (eIF2α) phosphorylation (86). This suggests that targeting STAT3 may be a viable strategy to rebalance protein turnover in cachectic muscle. Moreover, excessive STAT3 activity disrupts skeletal muscle regeneration by impairing satellite cell differentiation, proliferation, and self-renewal, ultimately leading to regenerative failure, premature differentiation, and age-associated muscle decline (86–88). Collectively, these mechanisms highlight STAT3’s central role in promoting muscle atrophy through enhanced proteolysis, dysregulated autophagy, and impaired regenerative capacity, key drivers of cancer cachexia progression.

Mitochondrial dysfunction further amplifies muscle degeneration and is increasingly recognized as a critical feature of cachexia-associated muscle wasting (89). STAT3 impairs mitochondrial biogenesis and function, leading to energetic deficits and contributing to muscle fatigue and weakness (88). Pharmacological inhibition of the JAK/STAT3 pathway has been shown to reverse myotube atrophy and restore mitochondrial protein levels in murine models of colorectal cancer cachexia (90) Additionally, IL-6 signaling through the gp130 receptor regulates mitochondrial quality control in skeletal muscle. During Lewis lung carcinoma (LLC)-induced cachexia, gp130-STAT3 signaling activates p38 MAPK, which in turn stimulates FOXO3 and Atrogin-1 expression, promoting muscle degradation (91, 92). STAT3 also exhibits immunomodulatory properties by engaging the PI3K/Akt axis, indicating potential crosstalk between inflammatory and metabolic pathways in cachectic muscle (93). Together, these findings highlight STAT3’s critical role in muscle wasting by integrating mitochondrial dysfunction, protein degradation, and metabolic disturbances, thereby reinforcing its potential as a therapeutic target for mitigating cancer cachexia.

The interplay between STAT3 and other muscle-wasting signaling pathways further exacerbates muscle atrophy. STAT3 upregulates myostatin, a potent inhibitor of muscle growth, which activates Smad2/3 signaling to suppress the Akt/mTOR axis and reinforce proteasomal degradation via MuRF1 and Atrogin-1 (26, 76, 83, 84, 94) (**Figure 3F**). TGF-β, another activator of Smad2/3, synergizes with STAT3 to intensify muscle wasting and fibrosis, in part through IL-6 amplification (95, 96). Notably, STAT3 activity correlates with cachexia severity in mouse models overexpressing TGF-β1 in skeletal muscle, further emphasizing this pathological axis (97) (**Figure 3C**). Synchronously, hypoxia-inducible factor 1α (HIF-1α), which is elevated in tumor-bearing conditions (98, 99), collaborates with STAT3 to shift muscle metabolism from oxidative phosphorylation to glycolysis (**Figure 3H**). This metabolic reprogramming impairs mitochondrial ATP production and contributes to muscle fatigue. Intriguingly, tumor-derived exosomal miR-183-5p activates both HIF-1α and STAT3, establishing a link between hypoxic signaling and enhanced proteolysis (100). The integration of these inflammatory, catabolic, and metabolic pathways underscores STAT3’s pivotal role in the rapid loss of muscle mass and function characteristic of advanced cancer cachexia.

### STAT3 in adipose loss

Cancer cachexia is marked not only by progressive skeletal muscle wasting but also by profound adipose tissue atrophy. Emerging evidence identifies STAT3 as a central mediator of these metabolic disturbances. In the C26 mouse model of cancer cachexia, elevated levels of STAT3 and its activated form (pY705-STAT3) have been observed in white adipose tissue, implicating this pathway in adipocyte dysfunction and fat loss (101). STAT3 activation drives lipolysis and systemic metabolic imbalance by promoting the breakdown of triglycerides and impairing lipid storage mechanisms, thereby exacerbating energy depletion characteristic of cachexia. IL-6 family cytokines, particularly leukemia inhibitory factor (LIF), initiate adipocyte lipolysis through a STAT3-dependent mechanism. Specifically, LIF-induced STAT3 activation upregulates adipose triglyceride lipase (ATGL) and its coactivator CGI-58, facilitating triglyceride hydrolysis (57). Concurrently, STAT3 suppresses peroxisome proliferator-activated receptor alpha (PPARα), a key transcription factor involved in lipid uptake and storage, thereby reducing lipogenesis and worsening adipose tissue dysfunction (59). Inhibiting the JAK/STAT3 pathway in murine models significantly attenuates lipolysis and prevents adipose tissue wasting, highlighting the therapeutic potential of STAT3 blockade in cancer cachexia (57). Moreover, STAT3 activation correlates with elevated lactate dehydrogenase (LDH) levels and diminished adiponectin production in adipocytes, further contributing to systemic metabolic disruption (102). Beyond white adipose tissue, STAT3 also regulates brown adipose tissue (BAT) homeostasis. Constitutive activation of STAT3 enhances brown fat thermogenesis and energy expenditure, as demonstrated by the reversal of obesity in TYK2 knockout mice through increased BAT differentiation (103). Conversely, STAT3 inhibition boosts the expression of uncoupling protein 1 (UCP1) and improves mitochondrial function in brown adipocytes, suggesting its dual role in modulating both lipolytic activity and thermogenic capacity (104).

STAT3’s regulatory role in adipose tissue wasting is further compounded by its interaction with the AMPK and Wnt/β-catenin pathways. While AMPK activation generally supports energy homeostasis by promoting fatty acid oxidation, its chronic stimulation in cachexia leads to destabilization of the AMPK complex in adipocytes, aggravating lipid depletion and energy imbalance (105, 106). Notably, systemic delivery of an AMPK-stabilizing peptide in tumor-bearing mice preserved adipose tissue mass and mitigated body weight loss without affecting tumor growth, underscoring the therapeutic relevance of modulating AMPK in concert with STAT3 signaling (106). Simultaneously, Wnt/β-catenin signaling, traditionally known for its role in adipogenesis and adipocyte differentiation, may intersect with STAT3 to regulate lipid turnover and adipocyte browning (107). Although this interaction remains under active investigation, preliminary findings suggest that Wnt/β-catenin-STAT3 crosstalk may influence adipocyte plasticity and energy metabolism, presenting another potential target for intervention. Collectively, these findings underscore STAT3’s multifaceted role in mediating adipose tissue atrophy in cancer cachexia through its regulation of lipolytic enzymes, suppression of lipogenesis, impairment of mitochondrial function, and modulation of key metabolic pathways. Targeting STAT3 and its interacting partners represents a promising strategy to alleviate the systemic metabolic derangements that characterize this debilitating syndrome.

### STAT3 and anorexia

Cancer cachexia-associated anorexia represents a critical clinical challenge, affecting up to 60% of patients with advanced malignancies (108). This condition significantly diminishes quality of life, compromises treatment efficacy, and correlates with poor survival outcomes (109, 110). Central to the regulation of appetite in this context is the transcription factor signal transducer and activator of transcription 3 (STAT3), which integrates peripheral metabolic and inflammatory signals in the hypothalamus. Leptin, a hormone secreted by adipose tissue, regulates energy homeostasis by binding to the long isoform of the leptin receptor (LepRb) expressed in the arcuate nucleus (ARC) of the hypothalamus. This interaction activates the JAK2-STAT3 signaling cascade, which modulates feeding behavior by acting on two distinct neuronal populations (111, 112). STAT3 enhances the activity of pro-opiomelanocortin (POMC) neurons, promoting satiety through the production of α-melanocyte-stimulating hormone (α-MSH) (113, 114), while concurrently inhibiting agouti-related peptide (AgRP) neurons that stimulate appetite (115). As part of a negative feedback mechanism, STAT3 upregulates suppressor of cytokine signaling 3 (SOCS3), which inhibits further JAK2-STAT3 signaling and contributes to leptin resistance (116). Functional studies underscore the physiological relevance of this pathway. Mice lacking STAT3 in POMC neurons develop obesity due to impaired satiety signaling, whereas constitutive STAT3 activation in AgRP neurons suppresses food intake (115). These findings illustrate the critical role of STAT3 in maintaining energy balance and suggest that its dysregulation can lead to pathological anorexia or hyperphagia depending on context.

Beyond leptin signaling, STAT3 is also activated by pro-inflammatory cytokines such as TNF-α (117) and CNTF (118), both of which suppress food intake through hypothalamic STAT3-dependent mechanisms (119). Moreover, STAT3 may mediate crosstalk between leptin and insulin signaling in the hypothalamus, though this interaction remains incompletely understood (120). Chronic overexpression of SOCS3, a direct STAT3 target, has been implicated in leptin and insulin resistance (113, 121), highlighting the importance of tightly regulated STAT3 activity for metabolic homeostasis. Leptin resistance is particularly relevant in cancer cachexia, where elevated leptin levels fail to restore appetite due to SOCS3-mediated inhibition of LepRb signaling (122, 123). This dysregulation is exacerbated in obesity-associated cancers, where both systemic inflammation and leptin resistance contribute to anorexia. Concurrently, pro-inflammatory cytokines activate the NF-κB pathway, which acts synergistically with STAT3 to enhance transcription of anorexigenic mediators and promote hypothalamic inflammation (124, 125).

In addition to inflammatory and hormonal signaling, STAT3 modulates nutrient-sensing pathways. It has been shown to inhibit AMP-activated protein kinase (AMPK), a key promoter of hunger, and potentially stimulate mechanistic target of rapamycin (mTOR) signaling, both of which suppress appetite during inflammatory states (126, 127). Moreover, recent studies implicate STAT3 in the anorexigenic GDF15-GFRAL signaling axis. Growth differentiation factor 15 (GDF15), elevated in several cancer types, signals through its receptor GFRAL in the area postrema and nucleus of the solitary tract (NTS), regions involved in nausea and appetite regulation. STAT3 activation may converge with GDF15 signaling in these brainstem nuclei, amplifying the anorectic response (128, 129). Altogether, these findings position STAT3 as a central node in the neuroimmune circuitry underlying cancer cachexia-associated anorexia. Through its integration of leptin, cytokine, and nutrient-sensing pathways, STAT3 contributes to appetite suppression and energy imbalance. Targeting STAT3 or its regulatory partners, such as SOCS3, may offer a promising therapeutic strategy for alleviating anorexia and improving outcomes in cachectic patients.

### STAT3 in immune modulation

Dysregulation of immune checkpoints is a well-established mechanism by which many cancers evade immune surveillance, thereby facilitating tumor progression (17). A growing body of evidence implicates STAT3 as a central regulator of this immunosuppressive network. Elevated STAT3 activity, frequently driven by pro-inflammatory cytokines such as IL-6 (18, 130), promotes immune escape through the transcriptional upregulation of key immune checkpoint molecules, including CTLA-4 (17), programmed cell death protein 1 (PD-1) (131), and its ligands PD-L1 and PD-L2 (132, 133). This STAT3-mediated enhancement of immune checkpoint expression contributes to tumor immune evasion and may help explain the limited efficacy of immune checkpoint blockade therapy in end-stage cancer patients, particularly those suffering from cancer cachexia (134, 135). Beyond checkpoint regulation, STAT3 profoundly alters T cell dynamics within the tumor microenvironment (TME). It promotes the differentiation of CD4^+^ T cells into regulatory T cells (Tregs), which facilitate immune tolerance, while simultaneously impairing the generation and cytotoxic function of CD8^+^ cytotoxic T lymphocytes (CTLs), the primary effectors of anti-tumor immunity (136). At the same time, STAT3 activation enhances the differentiation, expansion, and immunosuppressive activity of myeloid-derived suppressor cells (MDSCs) (137), further dampening adaptive immune responses and contributing to systemic inflammation. Emerging evidence highlights that STAT3 hyperactivation occurs not only in tumor cells but also in immune cells infiltrating the tumor microenvironment (TME) (138), affecting a broad spectrum of immune populations, including macrophages (139), T cells (140, 141), NK cells (142), and dendritic cells (143). This widespread STAT3 activity reshapes the immune landscape to favor tumor progression and contributes to the development of cancer cachexia.

In cachexia-prone malignancies, the TME is enriched with pro-inflammatory cytokines such as IL-6, TNF-α, IL-1β, and IFN-γ, secreted by both tumor and immune cells including macrophages, MDSCs, and T cells (4, 144). These cytokines promote chronic systemic inflammation and metabolic dysfunction, driving catabolic processes in skeletal muscle and adipose tissue. Within this inflammatory milieu, STAT3 plays a key role in skewing macrophage polarization toward the M2 phenotype (145, 146), which, although traditionally viewed as anti-inflammatory (147), contributes to tumor immune evasion by producing immunosuppressive cytokines such as IL-10 and TGF-β (148, 149). These cytokines, in turn, sustain a suppressive TME and reinforce STAT3 signaling, forming a self-perpetuating loop that fuels both tumor growth and cachexia-associated wasting (150). Importantly, preclinical studies have demonstrated that blockade of the IL-6/STAT3 axis restores anti-tumor immune responses by relieving T cell suppression and enhancing adaptive immunity (151). These findings underscore the pivotal role of STAT3 in orchestrating immune evasion and systemic catabolism, positioning it as a promising therapeutic target for addressing both cancer progression and the immunometabolic dysfunctions characteristic of cancer cachexia.

### Regulatory networks and post-transcriptional modulation: role of microRNAs

MicroRNAs (miRNAs) serve as key post-transcriptional regulators of STAT3 and its associated pathways. For instance, miR-203 targets SOCS3, indirectly enhancing STAT3 signaling, while miR-183-5p simultaneously activates Smad3 and STAT3, promoting muscle degradation via upregulation of Atrogin-1 and MuRF1 (100, 152). Additionally, pancreatic cancer-derived exosomal miRNA let-7b-5p activates STAT3/FOXO1 signaling, exacerbating insulin resistance and muscle wasting (153). These findings underscore the critical role of miRNAs in modulating STAT3 activity and amplifying its cachectic effects (**Figure 3I**).

## Therapeutic perspectives

**Figure 4:**
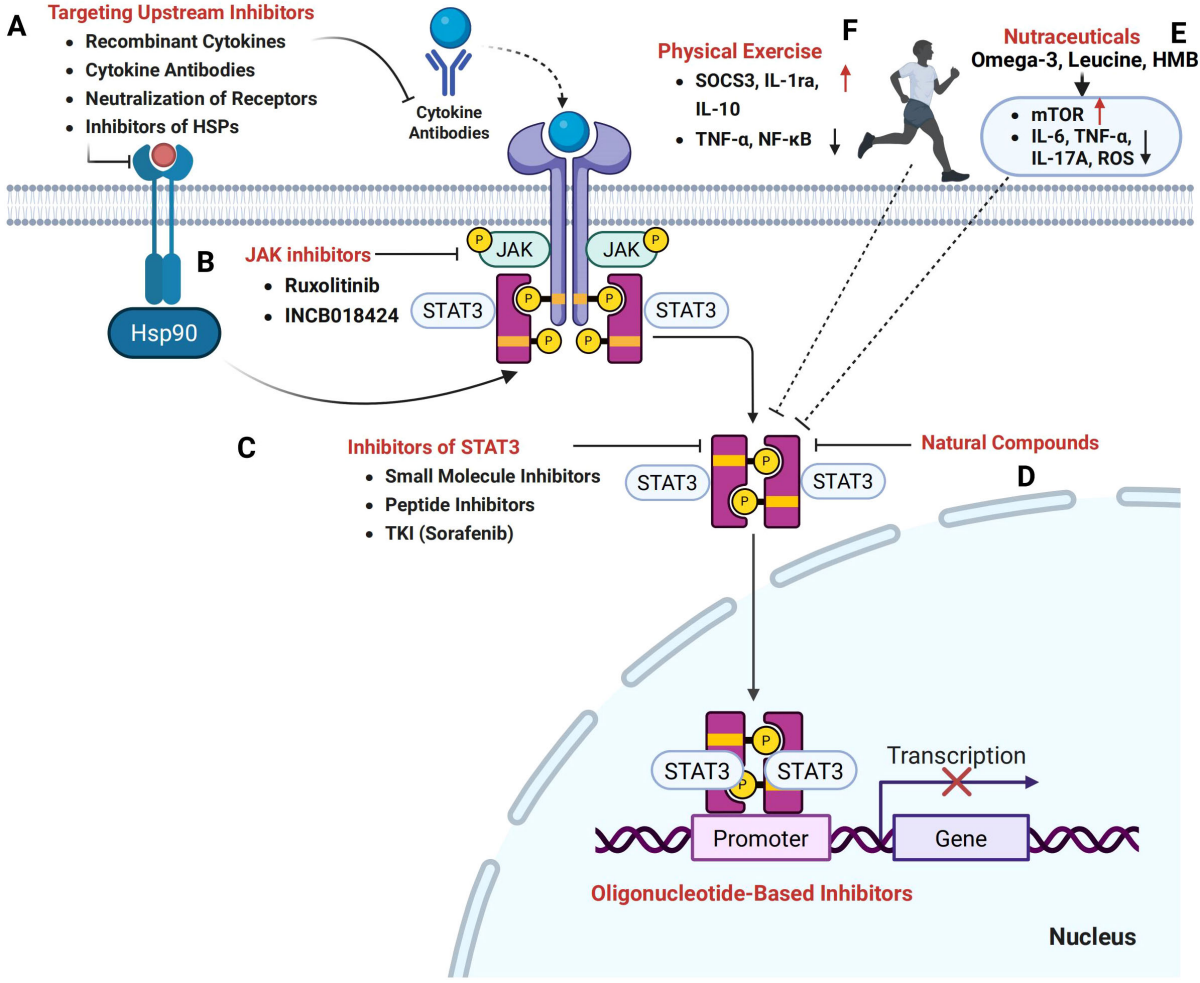
Therapeutic potential of targeting the STAT3 signaling pathway in cancer cachexia. **(A)** Therapeutic strategies aimed at upstream inhibitors, including recombinant cytokines, cytokine antibodies, receptor neutralization, and inhibitors of heat shock proteins (HSPs), hold promise in modulating STAT3 activation and mitigating cachexia. **(B)** Janus kinase (JAK) inhibitors, such as Ruxolitinib and INCB018424, effectively inhibit the JAK/STAT3 pathway. **(C)** Direct STAT3 inhibition can be achieved through small molecule inhibitors, peptide inhibitors, and tyrosine kinase inhibitors (TKIs) such as Sorafenib, which prevent STAT3 dimerization and nuclear translocation. **(D)** Natural compounds, including plant-derived phytochemicals, also target the STAT3 signaling axis. **(E)** Nutritional and metabolic modulators, particularly nutraceuticals, have the potential to influence STAT3-mediated pathways and restore the balance between protein synthesis and degradation. **(F)** Furthermore, exercise interventions targeting STAT3 signaling offer a promising strategy to counteract the muscle wasting and metabolic imbalance characteristic of cancer cachexia.

Given its central role in orchestrating inflammation, metabolic dysregulation, and tissue catabolism, STAT3 represents a highly promising but complex therapeutic target in cancer cachexia. This section explores current and emerging strategies aimed at modulating STAT3 signaling through cytokine inhibition, pharmacological agents, physical exercise, nutritional modulation, and integrated multi-targeted approaches (**Figure 4**).

### Upstream cytokine blockade

The JAK-STAT3 pathway, predominantly activated by cytokines such as IL-6, IFNγ, LIF, and OSM (69, 154, 155), plays a central role in cancer cachexia pathogenesis. Among these, IL-6 is a major activator of STAT3, with neutralization strategies showing substantial therapeutic promise. Preclinical studies using anti-IL-6 or anti-IL-6 receptor (IL-6R) antibodies in tumor-bearing mice demonstrate attenuation of muscle atrophy, adipose tissue loss, and systemic inflammation (27, 41, 145–147). Clinically, tocilizumab, a humanized monoclonal antibody targeting IL-6R, has improved symptoms and prognosis in cachectic patients, with Phase II trials in lung cancer confirming its ability to alleviate anorexia and weight loss (149, 156).

Other IL-6 family cytokines, including oncostatin M (OSM) and leukemia inhibitory factor (LIF), also contribute to cachexia through STAT3 activation. OSM induces myotube atrophy, while muscle-specific deletion of the OSM receptor (OSMR) preserves muscle mass in preclinical models (32). LIF promotes hepatic metabolic dysfunction via STAT3, and liver-specific deletion of the LIF receptor ameliorates cachexia-related lipid abnormalities (62). Neutralizing antibodies against OSM and LIF have shown efficacy in preclinical studies, making them viable adjuncts or alternatives to IL-6 blockade (150). Further, inhibition of IL-17A and small-molecule blockade of STAT3 phosphorylation (e.g., AG490) reduce cachexia severity (33), reinforcing the need for multi-cytokine targeting strategies (**Figure 4A**). Such combinatorial approaches may be particularly relevant for malignancies with high cytokine redundancy, such as pancreatic ductal adenocarcinoma (PDAC).

### JAK inhibitors

Inhibiting Janus kinases (JAKs) upstream of STAT3 has emerged as another promising therapeutic strategy. Agents such as ruxolitinib and INCB018424 have shown preclinical efficacy in mitigating cachexia symptoms by suppressing STAT3 phosphorylation, normalizing cytokine/adipokine profiles, and reducing tissue wasting (54, 57, 157, 158). Notably, JAK inhibition also prolongs survival in murine models of cancer cachexia, possibly by dampening systemic inflammation and restoring metabolic homeostasis (159). However, JAK inhibitors may affect multiple downstream targets beyond STAT3, necessitating strategies to enhance tissue specificity and minimize immunosuppressive side effects (**Figure 4B**).

### Pharmacological inhibitors of STAT3

Direct inhibition of STAT3 presents a promising therapeutic strategy, offering greater specificity and efficacy in preclinical models of cancer cachexia. Small-molecule inhibitors such as STATTIC, C188-9, and napabucasin act by disrupting STAT3 dimerization, DNA binding, or phosphorylation (160). Notably, C188–9 significantly reduced phosphorylated STAT3 (pY705-STAT3) levels in skeletal muscle from both chronic kidney disease and C26 tumor-bearing mice, accompanied by preservation of muscle mass, grip strength, and myofiber size (25, 161, 162). These inhibitors also ameliorate muscle wasting by restoring mitochondrial function, reducing proteolysis, and improving systemic metabolic parameters (15, 163–166). In addition to intrinsic STAT3 activation, tumor-associated macrophages (TAMs) exacerbate muscle degeneration by secreting pro-inflammatory cytokines, particularly IL-1α and IL-6, which activate STAT3 signaling in muscle fibers. Pharmacological blockade of macrophage-derived cytokines or direct inhibition of STAT3 in myofibers significantly attenuates muscle atrophy in pancreatic cancer models (139). Furthermore, targeting the HSP90/STAT3/FOXO1 signaling axis using inhibitors such as 17-DMAG and PU-H71 has been shown to suppress atrogene expression and mitigate muscle wasting (42). Moreover, the multi-kinase inhibitor sorafenib has demonstrated the ability to modulate STAT3 activity, preventing the accumulation of atrogin-1 and Pax7 in skeletal muscle, thereby improving functional capacity and reducing fatigue in tumor-bearing animals (167). In total, these findings underscore the therapeutic potential of STAT3-targeted interventions in cancer cachexia (**Figure 4C**).

Despite advances, several barriers hinder effective STAT3-targeted therapy. To enhance efficacy and minimize systemic toxicity, the development of tissue-specific delivery systems, such as nanoparticles or antisense oligonucleotides, is essential. First, tissue-specific actions of STAT3 necessitate precision in therapeutic targeting to avoid adverse effects (25). Second, off-target toxicity and resistance mechanisms limit the utility of current inhibitors (15, 168, 169). Development of highly selective, delivery-optimized agents (e.g., nanoparticle-based siRNAs or antisense oligonucleotides) is urgently needed. Additionally, identifying reliable biomarkers for STAT3 activation and cachexia progression remains a challenge. Personalized therapeutic approaches based on patient-specific cytokine profiles, tumor types, and metabolic status will be crucial for optimizing outcomes. Future directions include exploring combination therapies and integrating pharmacologic treatments with exercise and nutrition. A comprehensive, multi-targeted approach centered on STAT3 inhibition holds promise to transform cachexia management and improve quality of life in cancer patients.

### Natural products targeting STAT3

Several natural compounds have shown promising potential in inhibiting STAT3 activity, and are currently being investigated for their anti-cachexia effects (**Figure 4D**, **Table 1**). These compounds exhibit anti-inflammatory, lipid-sparing, and muscle-preserving properties by targeting various mechanisms, including the inhibition of STAT3 activation and the downregulation of pro-inflammatory cytokines, which drive muscle degradation. For instance, alantolactone (28), ursolic acid (170), and brassinin (171) reduce pro-inflammatory cytokines (such as IL-6 and TNF-α), as well as COX-2, and directly inhibit STAT3 signaling, thus alleviating tissue wasting. Additionally, compounds such as atractylenolide I (172), saikosaponin D (173), cucurbitacin IIb (174), imperatorin (175), ginsenoside Rd (176), cryptotanshinone (177), and Z526 (178) have also demonstrated the ability to suppress STAT3 activation and mitigate body weight and muscle loss in cachectic models. Notably, alantolactone (28), atractylenolide I (172), imperatorin (175), ursolic acid (170, 179), and brassinin (171) have further emphasized the therapeutic potential of targeting STAT3 to prevent fat loss in cancer cachexia. While these natural compounds show encouraging preclinical efficacy in targeting STAT3 to alleviate cancer cachexia, further clinical studies are necessary to confirm their therapeutic potential and safety for use in treating this condition.

**Table 1:** Natural compounds in ameliorating cancer cachexia via targeting STAT3 signaling.

Natural Products	Cachexia Model	Administration	Mechanism	Functions	References
Alantolactone	C26 tumor-bearing cancer cachexia model	10 mg/kg, i.p., qd, 13d.	Targeting STAT3 and NF-κB	Ameliorating muscle wasting and lipolysis, anti- inflammation.	(28)
Ursolic acid	C26 tumor-bearing cancer cachexia model	100 mg/kg, gavage, qd, 14d.	Inhibiting STAT3 and NF-κB	Increasing body weight, muscle mass, epididymal fat and food intake. Reduction of inflammatory factors	(170)
Brassinin	HT-29 cells-xenograft cancer cachexia model	1 mg/kg, i.p., 3 times a week, 4 weeks.	Inhibition of STAT3	Inhibiting weight loss, skeletal wasting, fat atrophy and inflammatory cytokines.	(171)
Atractylenolide I	C26 tumor-bearing cancer cachexia model	25 mg/kg, i.p., qd, 18d	Targeting STAT3/PKM2/SNAP23 pathway.	Attenuating weight loss, anorexia, glycolysis effect, muscle wasting, and adipose degradation.	(172)
Saikosaponin D	C26 tumor-bearing cancer cachexia model.	2.4 mg/kg, gavage, qd, 16d.	Binding to SH2 domain of STAT3.	Muscle Preservation Improving loss of body weight.	(173)
Cucurbitacin IIb	C26 tumor-bearing cancer cachexia model.	2 mg/kg, i.p., qd, 14d.	Regulating IL-6/JAK/STAT3/FoxO Signaling Pathway	Alleviating skeletal muscle and epididymal fat loss.	(174)
Imperatorin	C26 tumor-bearing cancer cachexia model.	25 or 50 mg/kg, gavage, qd, 15d.	Binding to the SH2 domain of STAT3	Preventing body weight loss and wasting of multiple tissues, such as skeletal muscle, fat and kidney.	(175)
Ginsenoside Rd	LLC1 and CT26 tumor-bearing cancer cachexia model.	10 mg/kg, gavage, qd, 5 weeks	Inhibiting STAT3 nuclear translocalization	Suppressing muscle atrophy Reducing ROS levels and protecting mitochondrial integrity.	(176)
Cryptotanshinone	CT26 tumor-bearing cancer cachexia model.	20 or 60 mg/kg, gavage, qd, 10d.	Prevention of STAT3 transcriptional activity	Increasing epididymal fat, skeletal muscle and myocardial mass, ameliorating food intake, inhibiting tumor growth and inflammation.	(177)
Z526	C26 tumor-bearing mice treated with oxaliplatin.	2.5 or 5 mg/kg, gavage, qd, 15d.	Inhibiting the activation of STAT3 and NF-κB.	Mitigating body weight, fat and muscle loss, and reducing oxidative stress.	(178)

### Physical exercise targeting STAT3

A growing body of evidence from both preclinical and clinical studies indicates that multimodal exercise interventions can effectively mitigate STAT3-driven muscle atrophy in cancer cachexia by modulating inflammatory signaling and enhancing anabolic pathways. During exercise, skeletal muscle fibers release IL-6 via a TNF-independent mechanism, which promotes the systemic release of anti-inflammatory cytokines such as IL-1 receptor antagonist (IL-1ra) and IL-10, while concurrently suppressing pro-inflammatory mediators including TNF-α and NF-κB activity (180–183). This anti-inflammatory cytokine profile contributes to the attenuation of chronic inflammation that sustains aberrant STAT3 activation in skeletal muscle. Resistance training, in particular, plays a critical role by not only reducing IL-6-induced STAT3 phosphorylation but also by upregulating suppressor of cytokine signaling 3 (SOCS3), a negative feedback regulator that directly inhibits STAT3 signaling (184). Endurance exercise complements these effects by reducing circulating levels of TNF-α and IL-6 and by promoting mitochondrial biogenesis and oxidative metabolism (185), thereby preserving muscle mass and function. Interestingly, exercise-induced IL-6 also exerts context-dependent protective effects, counteracting the catabolic actions of systemic inflammatory cytokines such as TNF-α, likely through autocrine and paracrine mechanisms (186–189). All in all, these findings highlight the therapeutic potential of structured exercise interventions in modulating STAT3 activity, offering a promising, non-pharmacological strategy to ameliorate muscle wasting and improve clinical outcomes in cancer cachexia.

### Nutritional and metabolic modulators

Recent therapeutic strategies have increasingly focused on targeting the STAT3 pathway to mitigate the muscle and adipose tissue wasting characteristic of cancer cachexia. Among these strategies, nutritional and metabolic modulators, particularly nutraceuticals, have emerged as promising adjuncts to conventional therapies (**Figure 4E**). Notably, omega-3 polyunsaturated fatty acids (n-3 PUFAs), especially eicosapentaenoic acid (EPA) and docosahexaenoic acid (DHA), have demonstrated significant anti-inflammatory properties. These effects are primarily mediated through the downregulation of pro-inflammatory cytokine signaling upstream of STAT3. For instance, n-3 PUFAs have been shown to suppress levels of IL-6 and TNF-α in cancer patients (190), as well as reduce IL-17A-mediated inflammation (191) and IL-11 expression in hepatocytes during acetaminophen-induced hepatotoxicity (192). These cytokines are known activators of STAT3 and play critical roles in sustaining chronic inflammation and tissue catabolism.

Beyond their anti-inflammatory properties, n-3 PUFAs have potential applications in enhancing exercise recovery and preserving skeletal muscle mass and strength (193–195). Mechanistically, supplementation with n-3 PUFAs has been found to activate the mechanistic target of rapamycin complex 1 (mTORC1) pathway, reduce intracellular protein degradation, and promote mitochondrial biogenesis and function (196). Additionally, dietary n-3 PUFAs may protect against muscle mitochondrial oxidative stress and attenuate muscle wasting in chronic heart failure (197), likely by enhancing oxidative phosphorylation efficiency, increasing ATP production, and reducing reactive oxygen species (ROS) accumulation and muscle fatigue.

In tandem, other nutritional agents such as leucine and its metabolite β-hydroxy β-methylbutyrate (HMB) have also demonstrated anti-cachectic potential, partly through modulation of STAT3 activity. Leucine enhances mitochondrial biogenesis and activates mTOR signaling in skeletal muscle while simultaneously decreasing STAT3 phosphorylation and associated inflammatory signaling in tumor-bearing mice (198, 199). Moreover, HMB has been shown to reduce muscle proteolysis and prevent apoptosis of myonuclei by inhibiting both the ubiquitin–proteasome and autophagy–lysosome pathways (197, 200). These effects collectively support its therapeutic value in various forms of cachexia.

## Challenges and future directions

Despite significant progress in elucidating the role of STAT3 in cancer cachexia, several key challenges remain that hinder the development of effective therapeutic strategies. One major obstacle is the complexity of STAT3 signaling pathways. STAT3 mediates a broad array of cellular functions, including immune regulation, tumor progression, tumor inflammation, and metabolic reprogramming (15), with tissue-specific responses across various disease contexts (26, 43). Its multifaceted involvement makes it difficult to delineate the exact mechanisms by which STAT3 contributes to cachexia and to develop therapies that selectively disrupt its pathological actions. STAT3’s central role in maintaining immune homeostasis, particularly in anti-tumor immunity, further complicates therapeutic targeting, as systemic inhibition may result in unintended immunosuppressive effects (17, 165).

A second major challenge lies in the tissue-specific and sometimes opposing roles of STAT3. For instance, while it drives skeletal muscle wasting in cancer cachexia, it also regulates lipolysis in adipose tissue and metabolic dysfunction in the liver (25). Dissecting these tissue-specific functions is essential for designing targeted interventions that can suppress STAT3’s deleterious effects in muscle without impairing its roles in other organs. Additionally, the TME is intimately involved in STAT3-driven cachexia, as STAT3 regulates immune cell polarization, cytokine production, and metabolic crosstalk between tumor and host tissues (15, 201). Therapeutic targeting of STAT3 must therefore be carefully balanced to avoid inadvertently promoting tumor progression while alleviating cachexia.

Despite the identification of numerous STAT3 inhibitors, translating these agents into clinical practice remains challenging. Many compounds suffer from poor pharmacokinetics, including low stability, poor oral bioavailability, and limited tissue penetration. Off-target effects and insufficient specificity have limited the efficacy and safety profiles of several candidates in human trials (15, 169, 202–204). Furthermore, because STAT3 is ubiquitously expressed and involved in essential physiological processes, global inhibition carries the risk of side effects such as immunosuppression, hepatotoxicity, and impaired tissue repair (18, 205). These concerns underscore the need for the development of next-generation STAT3 modulators that are highly selective, stable, and capable of achieving tissue-specific delivery (206).

An additional barrier to effective STAT3-targeted therapies is the emergence of resistance mechanisms. Tumors can activate alternative signaling pathways, such as NF-κB (20), or PI3K/AKT (207, 208), to circumvent STAT3 inhibition. This suggests that STAT3 monotherapy may be insufficient in many clinical contexts. Combination strategies involving STAT3 inhibitors with agents such as chemotherapy, radiotherapy, targeted therapy, or immunotherapy are promising approaches to enhance efficacy and mitigate resistance (15, 17). Despite extensive efforts to target STAT3 in cancer therapy, cancer remains a major clinical challenge, even with the advent of novel treatment strategies (168). Another critical unmet need is the lack of reliable biomarkers for early detection and monitoring of STAT3 activation in cancer cachexia. Non-invasive biomarkers, such as circulating cytokines, or phosphorylated STAT3, could facilitate early diagnosis, enable stratification of patients most likely to benefit from STAT3-targeted therapies. Incorporating such biomarkers into clinical trial design will be essential to advance STAT3 inhibitors from bench to bedside (209).

Given the complexity of STAT3’s biological roles and its diverse effects depending on tumor type and disease stage, future therapeutic strategies should adopt a personalized medicine approach. Integration of genomic, transcriptomic, and single-cell data can help identify context-specific STAT3 targets, allowing for more precise intervention. Such strategies would also consider the patient’s tumor profile, metabolic state, immune function, and comorbidities. Personalized treatment paradigms are likely to represent the future direction of STAT3-based therapies in cancer cachexia. In summary, successful translation of STAT3-targeted therapies into clinical use for cancer cachexia will require a multifaceted approach: (1) comprehensive characterization of tissue-specific STAT3 mechanisms, (2) development of highly selective and bioavailable inhibitors, (3) rational combination with other therapeutic agents, (4) discovery and application of predictive biomarkers, and (5) implementation of individualized treatment plans. Addressing these challenges will be key to unlocking the full therapeutic potential of STAT3 modulation in managing cancer cachexia.

## Conclusion

This review underscores the critical role of STAT3 in the pathophysiology of cancer cachexia, a debilitating syndrome characterized by progressive muscle wasting, systemic inflammation, and metabolic dysregulation. Activation of STAT3 by pro-inflammatory cytokines such as IL-6 and HSPs fosters a catabolic environment that accelerates disease progression. Mechanistically, STAT3 signaling contributes to muscle degradation by upregulating muscle-specific E3 ubiquitin ligases while simultaneously suppressing protein synthesis. Furthermore, it exacerbates fat loss by promoting lipolysis and disrupting adipokine homeostasis in adipose tissue. Given its central role in cachexia pathogenesis, therapeutic targeting of the STAT3 pathway through small molecule inhibitors, monoclonal antibodies, or combination therapies presents a promising avenue for symptom management and improved patient outcomes. However, the intricate interplay between STAT3 signaling, tumor biology, and host metabolism remains incompletely understood, necessitating further research to refine therapeutic strategies and validate their clinical efficacy and safety. The heterogeneity of cancer cachexia, influenced by tumor type, disease stage, and individual metabolic profiles, further complicates treatment approaches, highlighting the need for personalized interventions. Additionally, the potential risks associated with prolonged STAT3 inhibition, including immune suppression and impaired tissue regeneration, must be carefully balanced against its therapeutic benefits. Advancing our understanding of STAT3-targeted interventions and translating these findings into clinical practice could significantly enhance quality of life and survival outcomes for cancer cachexia patients. Future research should prioritize the identification of predictive biomarkers, the optimization of combination therapies addressing both muscle wasting and metabolic dysfunction, and the integration of adjunctive strategies such as exercise and nutritional support. By bridging the gap between molecular research and clinical application, STAT3-targeted therapies hold the potential to revolutionize cachexia management, offering a more comprehensive and effective approach to improving patient care.
